# Tracing the origins of *Qigong* fever: Mao Zedong, Jiang Weiqiao, quiet sitting, and the *jingluo* system, 1914–1958

**DOI:** 10.1017/mdh.2026.10079

**Published:** 2026-10

**Authors:** Shanshan Gao

**Affiliations:** Department of History and Philosophy of Science, University of Cambridge, Free School Lane, Cambridge, UK

**Keywords:** Chinese medicine, Jiang Weiqiao, *jingluo*, Mao Zedong, *qigong*, Quiet sitting

## Abstract

This paper traces the origins of *qigong* fever by locating Mao Zedong’s early practice of quiet sitting in 1916 within an intellectual lineage linking Jiang Weiqiao, Yang Changji, and Mao’s classmates. Deeply rooted in the Neo-Confucian tradition of self-cultivation and the circulation of *jingluo*, Jiang’s quiet sitting method was reinterpreted through Japanese hygienic languages during the Republican period, and later reframed in terms of Pavlovian neurophysiology in Maoist China. In 1954, Jiang’s claim that his long-term *qigong* practice had verified the existence of *jingluo* coincided with Mao’s renewed support for Chinese medicine. By legitimising the existence of *jingluo* through *qigong* practice, the circulatory *jingluo* system was codified into national Chinese medicine textbooks as the discipline’s core theoretical foundation, thereby laying the institutional and epistemic groundwork for the post-Mao *qigong* fever.

## Introduction

*Qigong* (breath training), renowned for its emphasis on meditative quiet sitting ( *jingzuo* ) and/or coordinated bodily movements, emerged as ‘the most widespread form of popular religiosity in post-Mao urban China’.^1^ While indigenous dynamic practices of ‘guiding and pulling to circulate *qi*’ ( *daoyin xingqi* ) and sexual cultivation ( *fangzhong* ) – performed in standing, kneeling, or lying postures – date back to the Warring States period (475–221 BCE), the static practice of cross-legged quiet sitting traces its origins to Indian Buddhist meditation ( *dhyāna* ). This seated contemplative mode of self-cultivation was introduced to China during the Eastern Han period (25–220 CE) through Buddhist scriptures translated from Central Asia.^2^ With the institutional formation of Daoism in the second century CE, Daoist practitioners gradually integrated indigenous theories of *yin–yang* and five phases ( *wuxing* ), together with techniques of *qi* circulation and sexual cultivation, into the Buddhist discipline of quiet sitting. This synthesis eventually crystallized into the distinct Daoist traditions of visualization meditation ( *cunsi* ), which flourished in fourth-century Shangqing practice, and internal alchemy ( *neidan* ), which matured during the Tang and Song dynasties.^3^ In the syncretic intellectual landscape of the Song dynasty (960–1279), Confucian thinkers integrated Buddhist quiet sitting and Daoist internal alchemy into a distinct Neo-Confucian mode of self-cultivation. In this context, quiet sitting referred to a seated practice of physical stillness, regulated breathing, and mental concentration intended to cultivate the heart-mind ( *xin* ) and *qi.* ^4^

Scholars such as Nancy N. Chen and David A. Palmer have traced the origins of *qigong* from 1949 onward.^5^ This paper, however, locates its roots further back to Mao Zedong’s (1893–1976) early practice of quiet sitting in 1916. Palmer and many Chinese scholars have overlooked Mao’s engagement with this practice, taking his critical remarks on Jiang Weiqiao’s (1873–1958) *Yinshizi’s Method of Quiet Sitting* in his 1917 essay ‘A Study of Physical Cultivation’ as evidence against his participation.^6^ However, this paper argues that Mao’s critique should not be interpreted as a rejection of quiet sitting, but rather as a reflection of familiarity and intellectual engagement. This research demonstrates that Mao’s involvement in quiet sitting was shaped by the influence of his teacher and future father-in-law, Yang Changji (1871–1920), and was shared among close classmates, including Cai Hesen (1895–1931) and Zhang Kundi (1890–1930). The key evidence supporting this argument is drawn from the biography of Mao written by his friend and classmate Xiao San (1896–1983).

Understanding Mao as a *qigong* practitioner in his youth is crucial to comprehending the health policies of the Chinese Red Army, the Chinese Communist Party (CCP), and the People’s Republic of China (PRC) under his leadership, particularly their endorsement of Chinese medicine and the respect accorded to its practitioners, especially those specialising in *qigong* and acupuncture. This background helps explain why *qigong* practitioners and acupuncturists played a central role in formulating Chinese medicine’s basic theories and national textbooks between 1954 and 1958, ultimately resulting in the dominance of the circulatory *jingluo* (channels and network vessels) system within what is now known as ‘Traditional’ Chinese Medicine (TCM).^7^ A key figure in shaping this development was Jiang Weiqiao, a leading advocate of quiet sitting during both the Republican period and the early PRC.

In 1954, Jiang claimed that his long-term *qigong* practice had verified the existence of *jingluo.* This assertion, alongside Mao Zedong’s own encounters with quiet sitting and the lure of scientifically validating the existence of the *jingluo* system, led Mao to view Chinese medicine as a ‘great treasure house’ with the capacity to redefine the foundations of modern biomedicine. Mao’s endorsement fuelled nationwide research into the nature of *jingluo*, the extensive utilisation of acupuncture anaesthesia during the Cultural Revolution (1966–1976), and the subsequent surge of *qigong* fever that swept across the nation in its aftermath. By tracing the historical connections between Mao Zedong and Jiang Weiqiao, *qigong* and *jingluo*, this paper invites a critical reassessment of how political leadership, bureaucratic appointments, and nationalist ambition reshaped the nature of Chinese medicine.

## Jiang Weiqiao and *Yinshizi’s Method of Quiet Sitting*

The modern fascination with *qigong* can be attributed to its deep historical roots in Song Dynasty China, when Neo-Confucianism assimilated concepts and terminologies from Buddhism and Daoism and came to be widely embraced by literati and elites. Neo-Confucian scholars adopted ‘quiet sitting to purify the heart-mind’ and ‘sitting-in-forgetfulness’ practices, originally derived from Buddhism and Daoism religious traditions, into their daily routines as means of self-cultivation. Both Jiang Weiqiao and Mao Zedong were educated in Neo-Confucian thought in their youth and were shaped by its emphasis on *qi* and quiet sitting. In their respective efforts to modernise China and construct a scientifically grounded national identity, both figures sought to revive and reformulate traditional Chinese self-cultivation practices as a vital component of national revitalisation.

In his self-compiled chronological biography, Jiang Weiqiao described himself as frail and frequently ill from a young age, attributing his debilitated condition to the harmful effects of habitual masturbation.^8^ After years of ineffective medical consultations and treatments, around the age of eighteen (1890–1891), his father, who was well-versed in the *Book of Changes* ( *Yijing* ) and skilled in divination, introduced him to texts on the Neo-Confucian heart-mind cultivation and the internal practice of Microcosmic Orbit, as recorded in the *Collected Explanations of Medical Prescriptions* ( *Yifang jijie* ), authored by the Qing dynasty Neo-Confucian physician Wang Ang (1615–1695). Upon reading these works, Jiang reported experiencing a sudden sense of enlightenment and began practising the method, which led to what he described as ‘a marked improvement in my health’.^9^

The Microcosmic Orbit is a classical Daoist internal alchemy quiet sitting technique that directs concentrated awareness through a continuous circuit along the body’s two central vessels ( *mai* ). The *Du* (Governing) vessel ascends the spine from the perineum to the crown of the head, while the *Ren* (Controlling) vessel descends along the front of the body from the mouth down to the perineum, together forming a closed midline loop of circulation, as documented in the *Collected Explanations of Medical Prescriptions*:

To begin, still the thoughts, allowing body and mind to become clear and calm. Sit facing east in a cross-legged posture, breathing gently and steadily, form the samādhi mudrā below the navel. Knock the teeth thirty-six times to gather the body’s spirit. Perform the ‘Red Dragon Stirs the Sea’ (stirring the tongue within the mouth) thirty-six times both internally and externally. Rotate the eyes in coordination with the direction of the tongue’s movement.

Press the tongue against the upper palate, quiet the heart-mind, and count breaths. After completing three hundred and sixty [inhalation-exhalation] cycles, wait for the divine water (saliva) to accumulate in the mouth, then stir it several times. Then, using the ‘four-character formula’, begin at the *Ren* vessel by contracting the anus, then move focus to the tailbone. With concentrated intent, direct awareness upward along the spinal nodes towards the Middle Gate, gradually accelerating the pace.

With eyes closed and gazing upward, inhale through the nose without exhaling. Propel the awareness past the Jade Pillow (occipital bone), with a slight forward motion of the eyes, move the focus directly to *Kunlun* (crown of the head). Descend via the Magpie Bridge (tongue), divide the saliva, downward through the *Chonglou* (trachea), entering the *Li* Palace (heart), and finally reaching the *Qihai* (*Kan* Palace, also known as *Dantian* at the lower abdomen).

Pause briefly, then repeat the entire process three times. The saliva in the mouth should be swallowed in three separate portions. This technique is also known as Reversing the Heavenly River’s Flow.^10^

In the above method for unblocking the *Ren–Du* vessels, the key element is not *qi* but rather saliva, or essence (including sperm); this helps explain why Jiang adopted the practice to address the frailty he attributed to habitual masturbation. Beginning at the age of twenty-eight (1900), Jiang committed himself earnestly to the practice of Microcosmic Orbit.^11^ After eighty-five consecutive days of uninterrupted practice, he reported sensing the circulation of his *Ren–Du* vessels, accompanied by episodes of spermatorrhoea occurring every other day. From that point onward, he sustained an unbroken commitment to this quiet sitting method.^12^

In 1902, Jiang relocated to Shanghai, where he joined the Commercial Press and undertook the translation of Japanese textbooks. On 2 July 1913, he began reading Okada Tokujirō’s (1872–1920) *Okada’s Method of Quiet Sitting.* The next day, he recorded in his diary: ‘I plan to integrate our nation’s quiet sitting method with *Okada’s Method of Quiet Sitting* to author a new book’.^13^ He titled this new work under his alternative name, Yinshizi. *Yinshizi’s Method of Quiet Sitting* was issued by the Commercial Press in October 1914.^14^ In his 1917 preface, Jiang traced the ancient origins of quiet sitting and reflected on its precarious status in modern China:

The method of quiet sitting corresponds to internal cultivation in ancient times. In antiquity, the arts of health preservation comprised both external and internal cultivation. While medical treatments such as pharmacological remedies and acupuncture were employed to address diseases after their onset, health preservation focused on preventing disease before it occurred through the combination of external physical exercise and internal meditative cultivation.

Over time, however, the authentic transmission of cultivation methods was lost. Practitioners of external cultivation often lacked refinement and education, while internal cultivation became the domain of Daoist adepts, who conflated their methods with esoteric doctrines including *yin–yang*, five phases, *kan* and *li* palaces, and alchemical substances lead and mercury. Consequently, these methods became enveloped in mysticism and fell into disrepute among the educated elite.

In writing this book, I have dispelled the mystical and esoteric connotations traditionally associated with quiet sitting, instead offering explanations grounded in psychology and physiology. Every statement is based on personal experience and empirical observation. Regarding the correct method of breathing, I have also incorporated aspects of Okada’s teachings.^15^

Jiang’s teaching centres on two fundamental principles: (1) the correct method of sitting, and (2) the correct method of breathing. During the Republican era, many Japanese publications were translated and published in China under the names of Chinese translators or publishers, often with limited or no acknowledgement of the original sources.^16^ Chi-Yang Liao suggests that Jiang’s *Yinshizi’s Method of Quiet Sitting* was one such case, essentially a Chinese reinterpretation of *Okada’s Method of Quiet Sitting.* Hsiu-fen Chen further highlights the striking similarities between the two works, particularly in their sitting postures, breathing techniques, as well as the books’ content and stylistic presentation.^17^ By modernising and legitimising traditional self-cultivation practices through the language and authority of contemporary science, *Yinshizi’s Method of Quiet Sitting* achieved considerable popularity in China, evidenced by twelve reprints between 1914 and 1918, followed by five additional reprints of a revised edition between 1933 and 1941. Leveraging the success of this book, Jiang emerged as a leading authority on quiet sitting in Republican China, especially within educational and intellectual circles.

Beginning in 1916, Jiang assumed the position of policy advisor at the Ministry of Education. During this period, his vigorous promotion of quiet sitting gained significant traction among student communities, ranging from elite institutions like Peking University to numerous normal and secondary schools, especially in provinces such as Jiangsu, Zhejiang, and Hunan. The influence was especially pronounced in Hunan. At Changjun Middle School, for example, all students were required to perform deep breathing exercises during the first morning assembly. At the Hunan Provincial First Normal School, where Yang Changji taught and Mao Zedong studied from 1914 to 1918, quiet sitting was taught in self-cultivation classes. Mao had previously attended the Hunan Provincial Fourth Normal School from spring 1913 to spring 1914, where the practice of quiet sitting and deep breathing was likewise implemented. Chen Runlin (1879–1946), who served as both principal and freshman class instructor during Mao’s time at the school, was a strong proponent of quiet sitting among students.^18^

Jiang converted to Buddhism and began studying under Dixian (1858–1932) in 1915. From this point onward, he adopted the Tiantai Buddhist meditation method of samatha-vipassanā (calming and contemplation), drawing on canonical Buddhist texts including *Beginner’s Zhiguan* ( *Tongmeng zhiguan* ) and *Stages of Meditation in the Perfection of Dhyāna* ( *Shichan boluomi cidi famen* ). In 1922, Jiang compiled and published the *Supplement to Yinshizi’s Method of Quiet Sitting* ( *Yinshizi jingzuofa xubian* ), which was reprinted four times within the first year and five additional reprints between 1933 and 1941.^19^

In 1926, Jiang turned to the study of the Eighteen Methods of Shingon Buddhism, one of the major esoteric schools of Buddhism in Japan, under the guidance of Ācārya Chisong (1894–1972). Following this shift, he redirected his meditative focus to the Central Palace, a locus situated at the intersection of the chest and abdomen. As Jiang reported:

Within just a few days [of practicing the Eighteen Methods], my body underwent profound changes, and I succeeded in opening up (achieving unobstructed circulation through) the six vessels: *Yangqiao* (Yang Springing), *Yinqiao* (Yin Springing), *Yangwei* (Yang Linking), *Yinwei* (Yin Linking), *Chong* (Thoroughfare), and *Dai* (Girdling).^20^

These six vessels, together with the *Ren* and *Du*, comprise the Eight Extraordinary Vessels within the *jingluo* system. Although Jiang’s first publication dismissed earlier Chinese quiet sitting texts as ‘entire pages saturated with discourses on *yin–yang* and five phases’,^21^ each stage of his quiet sitting practice ultimately came to align closely with traditional medical concepts, including the Microcosmic Orbit (*Ren–Du* circulation) and the remaining six Extraordinary Vessels, all situated within the *jingluo* framework and grounded on the *yin–yang* and five phases theories.

In addition to his 1914 *Yinshizi’s Method of Quiet Sitting*, Jiang collated, revised, or wrote prefaces for multiple Japanese works on quiet sitting translated into Chinese and published between 1916 and 1920 by the Commercial Press, where he was employed. These included a Chinese edition of *Okada’s Method of Quiet Sitting*, which he translated himself.^22^ Together, these publications helped generate a quiet sitting fever in Republican China, which in turn gave rise to numerous original works by indigenous practitioners^23^ and the establishment of several hospitals, sanatoriums, and clinics in the 1920s and 1930s that utilised quiet sitting as a therapeutic method for conditions such as spermatorrhoea, erectile dysfunction, and functional gastric discomfort.^24^ The popularity of quiet sitting also contributed to the rise of redemptive societies in Republican China, most notably the Tongshan and Tiande societies.^25^

## Mao Zedong’s practice of quiet sitting

Jiang Weiqiao and his *Yinshizi’s Method of Quiet Sitting* exerted a profound influence on the young Mao Zedong, primarily through Mao’s teacher and future father-in-law, Yang Changji, a devoted practitioner of quiet sitting. From February 1914 until Yang’s death in January 1920, Mao studied under him with deep dedication. While teaching ethics and self-cultivation at the Hunan Provincial First Normal School, Yang profoundly shaped young Mao’s intellectual outlook and way of life. After Yang was appointed professor at Peking University in 1918, he helped Mao secure a position at the university library and allowed him to live in his home.^26^ Following Yang’s death, Mao married his daughter, Yang Kaihui (1901–1930), in the winter of 1920. In his 1936 interview with Edgar Snow (1905–1972), Mao reflected on his youth, admitting that he ‘was then an idealist’.^27^ He acknowledged the deep influence of his mentor Yang Changji, who ‘was an idealist with high moral character’, and ‘made the strongest impression’ on him during his formative years.^28^ Mao described Yang as ‘magnanimous and profound, utterly beyond comparison’, and frequently visited his residence to seek instruction.^29^ Li Rui (1917–2019), who later examined Mao’s notebooks written under Yang’s supervision, remarked that he ‘found it difficult to distinguish between Mao’s own ideas and those of Professor Yang, the two seemed to meld into one’.^30^

Although Yang engaged deeply with Western ethical theories and spent ten years studying in Japan and Europe, his intellectual identity remained firmly grounded in the Neo-Confucian tradition.^31^ Owing to his profound reverence for Neo-Confucianism and perhaps also to his poor health, he was both a devoted practitioner and an ardent advocate of quiet sitting.^32^ A close examination of Yang’s private diaries from five different years^33^ reveals ten explicit references to the practice of ‘quiet sitting’ and ‘deep breathing’, underscoring their importance in his daily regimen.^34^ During his tenure as professor of ethics at First Normal, Yang adopted a selected and translated version of Friedrich Paulsen’s *System der Ethik* (*System of Ethics*) as his course textbook,^35^ but rendered ‘ethics’ as ‘self-cultivation’, a choice that reflected the Neo-Confucian moral lexicon.^36^ Yang’s ethics curriculum extended beyond Western moral philosophy to incorporate traditional Chinese self-cultivation practices, among which quiet sitting occupied a foundational place.

In his diary entries from November 1914, one month after the publication of Jiang’s widely acclaimed *Yinshizi’s Method of Quiet Sitting*, Yang composed two reflective questions under the thematic heading ‘Issues of Self-Cultivation’, titled ‘The Benefits of Quiet Sitting’ and ‘The Methods of Quiet Sitting’.^37^ In addition, he demonstrated a keen interest in hygiene and physical training methods introduced from Japan and Europe, such as fasting, cold-water bathing, and various forms of physical exercise.^38^ This intellectual disposition likely explains Yang’s appreciation of Jiang’s method of quiet sitting, which integrated modern scientific discourse and techniques with Neo-Confucian self-cultivation. While teaching at First Normal, Yang insisted that ‘every morning, during the first student assembly, all students should be instructed to practice deep breathing’. He further recorded in his diary: ‘During the experimentations over the past few days, it was observed that very few students were able to keep their mouths sealed. Henceforth, it should be strongly advocated that the mouth ought to be kept closed’.^39^ The author infers that these ‘experimentations’ most likely involved deep breathing or quiet sitting exercises. The emphasis on deep breathing during quiet sitting, particularly the instruction that ‘the mouth ought to be kept closed’, was precisely one of the core principles in *Yinshizi’s Method of Quiet Sitting.* ^40^

From the fall term of 1917 through the first term of 1918, Mao enrolled in Yang’s course on self-cultivation. During this period, he diligently studied the assigned textbook and produced over 12,000 characters of detailed annotations and commentaries.^41^ Mao’s essay, titled ‘The Energy of the Heart-mind’,^42^ earned high praise from Yang, who, as Mao later recalled, ‘from his idealist viewpoint, he gave me a mark of 100 for it’.^43^ On September 16, 1917, Mao further elaborated his theory of ‘Heart-mind Energy’ in discussions with his close friends and classmates Cai Hesen and Zhang Kundi, asserting that ‘when one’s heart-mind energy and physical strength are united in action, there is no task that cannot be accomplished’.^44^ Under Yang’s influence, Mao and his companions adopted a Neo-Confucian lifestyle, upholding the principle of ‘preserving the heavenly *li* and extinguishing human desires’. Mao claimed to have no interest in romantic relationships, and members of his inner circle avoided discussing trivial or personal matters. Instead, Mao and his friends ‘preferred to talk only of great matters—the nature of man, of human society, of China, the world, and the universe!’^45^

According to Mao’s friend and classmate Xiao San, quiet sitting occupied an important place in the Neo-Confucian way of life that the young Mao Zedong embraced:

Mr. Yang Huaizhong’s^46^ influence on his students extended beyond the classroom, it shaped their lifestyle and disciplined way of life. […] He abstained from breakfast, practised deep breathing, advocated quiet sitting, and took cold-water baths throughout the year without pause, even in winter. Passionate youths like Mao Zedong, Cai Hesen, and others followed his example. For roughly one or two years, they too refrained from eating breakfast. During one summer vacation, Mao, Cai, and their comrade Zhang Kundi lived together at the Aiwan Pavilion on Mount Yuelu, across the river from Changsha, where they studied, rested, and cultivated. […] There, every early morning, they would practice quiet sitting on the mountain, followed by cold-water baths in the pond or river. They maintained this regimen after returning to school, persisting into November despite the onset of winter. […] On that mountain, against this backdrop, Mao Zedong and his comrades followed a daily routine devoted entirely to physical exercise, quiet sitting, reading books and newspapers, discussion, and contemplation.^47^

As the first biography of Mao authored by a Chinese writer after Edgar Snow’s *Red Star Over China*, Xiao’s account was widely regarded as both reliable and authentic. A close friend and classmate of Mao during his youth, Xiao drew not only on his personal recollections but also on interviews with individuals who had known Mao in his early years, thereby lending his record of Mao’s quiet sitting a considerable degree of historical credibility.^48^ The present article argues that the above episode of Mao, Cai, and Zhang’s practice of quiet sitting most likely took place during the latter half of 1916.^49^ Wang Guangxia (1894–?), who studied alongside Cai at the Hunan Higher Normal School from 1915 to 1917 and shared a dormitory with him, was himself an enthusiast of quiet sitting. He later recalled Cai’s unwavering dedication to the practice during that period:

Thus, he purchased a copy of *Yinshizi’s Method of Quiet Sitting* and practised quiet sitting three times a day: morning, noon, and evening. No matter how hectic or pressing the circumstances, he never once broke this routine.^50^

Zhang Kundi’s diary also recorded Cai Hesen’s daily routine during the summer vacation of 1917 in Changsha:

Rising at 4:30 a.m., he would climb to the summit of Mount Tianma to practice quiet sitting and physical exercise until 9:00 a.m., before descending. […] After lunch, around 4:00 p.m., he would either engage in quiet sitting, physical exercise, or take a walk.^51^

Given that Cai was a devoted practitioner of Jiang’s *Yinshizi’s Method of Quiet Sitting*, it is reasonable to infer that Mao likewise adopted Jiang’s method when practising quiet sitting together with Cai and Zhang in 1916.

Xiao San’s recollections of Mao’s quiet sitting practice first appeared in his serialised article ‘Comrade Mao Zedong’s Youth’, published between 1946 and 1947 in the CCP’s Jin–Cha–Ji Border Region.^52^ The work was later issued as standalone volumes in November 1947.^53^ During this period, the dynamics of the second phase of the Chinese Civil War (1945–1949) were shifting rapidly in the CCP’s favour, as Communist control expanded swiftly across newly occupied regions, particularly in Northeast and North China. This created an urgent demand for printed materials that could serve propagandistic purposes. In response, Xiao’s memoirs of Mao were compiled into at least seventeen separate publications between 1947 and 1949, reprinted multiple times, and widely circulated.^54^ The present study examined and compared six versions of Xiao’s biography of Mao published before 1950, in which the accounts describing Mao’s quiet sitting practice remain consistent in detail.^55^ In contrast, all three editions published after 1950 deleted the narrative of Mao’s quiet sitting practice entirely.^56^

## Mao Zedong’s advocacy of physical cultivation

Influenced by Yang Changji, who advocated an integrated approach combining internal quiet sitting with external physical exercise, and developed his own regimen under the name Seven-Section Cultivation ( *qiduan gong* ), both Mao Zedong and Cai Hesen engaged earnestly in practices that combined bodily movement with deep breathing and meditative focus. In April 1917, with Yang’s editorial support and recommendation, Mao published his first article, ‘A Study of Physical Cultivation’, in *New Youth* ( *Xin qingnian* ). In this essay, Mao invoked the well-known *Yinshizi’s Method of Quiet Sitting* as a counterpoint to his own proposed regimen, the Six-Section Physical Exercise ( *liuduan yundong* ), critiquing traditional sedentary meditation while articulating a dynamic vision of physical cultivation.

He began the article with the declaration: ‘The nation’s strength is in decline, the martial spirit has waned, and the physical constitution of the people grows increasingly frail and delicate’.^57^ He then introduced physical cultivation as the Western counterpart to the Chinese way of health preservation,^58^ setting forth his own theory of physical cultivation in contrast to the quietist traditions of Neo-Confucian thinkers Zhu Xi (1130–1200) and Lu Jiuyuan (1139–1193), as well as to the contemporary influence of Jiang Weiqiao’s teachings:

Master Zhu emphasised reverence ( *jing* ), while Master Lu emphasised stillness ( *jing* ). Stillness means remaining unmoving; reverence, though not involving motion, is likewise a state of stillness. As Laozi remarked, ‘Inaction is supreme’, and the Buddha too pursued tranquillity and stillness. Hence, the practice of quiet sitting has long been esteemed by the followers of Masters Zhu and Lu.

Recently, however, a figure known as ‘Yinshizi’ has been promoting a method of quiet sitting, extolling its miraculous efficacy and claiming that physical exercise is harmful to the body. While this may represent one possible path of self-cultivation, I do not presume to follow it myself. In my humble view, the essence of the universe lies in movement. When this universal principle is applied to human beings, it becomes what I call physical cultivation.^59^

He went on to lament his own frail constitution, explaining that this sense of inadequacy had led him to the study of hygiene and health preservation methods:

As human beings, we possess nothing but this body, this physical form containing our viscera and *luo* (network vessels). Though the methods of health preservation are many, their ultimate aim is simply to ensure the unobstructed circulation of *xuemai* (blood vessels).

During physical exercise, the heart-mind should be wholly focused on the bodily movement, eliminating all stray and distracting thoughts. Attention should be directed to the unobstructed circulation of *xuemai* , the contraction and relaxation of muscles, the articulation of joints, and the rhythmic flow of breath.^60^

Mao further likened his theory of physical cultivation to Zhu Xi’s teaching, bridging bodily practice and meditative focus:

Zhu Xi discussed the principle of maintaining single-mindedness without distraction, stating that one should concentrate solely on eating while eating, and solely on dressing while dressing. The same principle applies to physical exercise: one should devote one’s full, undivided attention to the movements themselves.

Having engaged in various forms of exercise, I found that they emphasised only external movements while neglecting the cultivation of the heart-mind. Therefore, I drew upon the strengths of each and devised a regimen of my own.^61^

Mao named his regimen the Six-Section Physical Exercise, as it consisted of six parts:

1. Hand exercise, sitting position: (…).
2. Foot exercise, sitting position: (…).
3. Trunk exercise, standing position: (…).
4. Head exercise, sitting position: (…)
  1. Massage with the hands (…)
  2. Free exercise. While keeping the general position of the head still, focus the mind to initiate movement of the skin and lower jaw, repeat five times.
5. Striking exercise, free-form position: (…).
6. Harmonising exercise, free-form position: (…)
  1. Deep breathing, repeat three times.^62^

Mao’s nomenclature for the Six-Section Physical Exercise was likely inspired by Yang’s Seven-Section Cultivation, as well as by Cai’s consistent practice of the Eight-Section Brocade ( *baduan jin* ),^63^ a health cultivation practice combined quiet sitting with massage that originated in the Song dynasty in its civil sitting form (known as the *wen baduan* ) and later evolved into a martial standing routine ( *wu baduan* ) that enjoyed widespread popularity from the late Qing through the Republican period.^64^ Following this lineage, although the main theme of Mao’s writings was to emphasise the importance of physical cultivation, thereby devaluing quiet sitting, his conception of exercise nonetheless diverged significantly from modern Western paradigms. Instead, Mao’s regimen remained deeply rooted in Neo-Confucian self-cultivation, combining an emphasis on meditative focus, heart-mind and viscera–*luo* theory, and the unobstructed circulation of *xuemai* , with traditional practices such as seated exercises, massage, intentional movement guided by mental concentration, and deep breathing. Mao’s advocacy of integrating meditative focus with coordinated bodily movements later served as a blueprint for the development of *qigong* in Maoist China.

Mao’s emphasis on *luo* and *xuemai* ^65^ did not derive from Jiang’s *Yinshizi’s Method of Quiet Sitting*, which borrowed heavily from modern scientific discourses and therefore avoided explicit reference to traditional Chinese medical concepts. Rather, Mao’s focus on ensuring the unobstructed circulation of *xuemai* was in fact more traditionally grounded than Jiang’s teachings in the 1910s and relied more directly on the classical *jingluo* system. This divergence may offer another reason for Mao’s dissatisfaction with Jiang’s quiet sitting method. Mao’s conception blended the traditional understanding of *luo* and *mai* with the anatomical notion of blood vessels, an interpretation that aligns closely with evidence from early *mai* manuscripts excavated since the 1970s from Mawangdui, Zhangjiashan, and Laoguanshan,^66^ as well as with the transmitted *jingluo* classics *Zhenjiu jiayijing* , *Huangdi neijing taisu* , and *Lingshu* . All of these transmitted sources stated explicitly that ‘the *mai* receives the blood and distributes it’, indicating that the term was understood in the sense of blood vessels.^67^ By contrast, during the Republican period, under the influence of Japanese acupuncture research, the mainstream reinterpretation of *jingluo* increasingly identified them with the nervous system rather than with blood vessels.^68^

During his 1936 conversation with Edgar Snow, Mao reflected with satisfaction on the physical cultivation of his youth, emphasising the enduring benefits it had brought him in terms of physical strength, discipline, and resilience:

We slept in the open when frost was already falling and even in November swam in the cold rivers. All this went on under the title of ‘body training’. Perhaps it helped much to build the physique which I was to need so badly later on in my many marches back and forth across South China, and on the Long March from Kiangsi to the Northwest.^69^

As the rising red star of the Communist movement, Mao chose not to disclose his quiet sitting practice to the American journalist. Yet he continued to engage with various forms of traditional health preservation throughout his later life. Notably, he adhered to the routine of ‘washing feet before sleep’, a regimen originally advocated by his fellow townsman Zeng Guofan (1811–1872) and consistently maintained by Jiang Weiqiao, from which Mao himself claimed to have ‘derived considerable benefit’.^70^ During the Yan’an period, Mao’s close associates Xie Juezai (1884–1971) and Xu Fanting (1893–1947) were also consistent practitioners of quiet sitting or meditation.^71^ In 1942, Mao received acupuncture, herbal remedies, and massage treatments for joint pain developed during the Long March (1934–1936). Among his physicians at that time was Lu Zhijun (1911–1999), who later became a leading advocate of acupuncture and *qigong* therapy in Maoist China. Benefiting from his close relationship with Mao, Lu served as the long-standing director of the China Academy of Chinese Medicine (1954–1978) and survived multiple political campaigns from the 1950s to the 1970s.^72^ According to Li Zhisui’s (1919–1995) memoir of Mao’s post-1949 life, Mao believed that ‘smoking is also a deep breathing exercise’, engaged in Daoist sexual cultivation practices, and asked his young companions to read the *Classic of the Pure Woman* ( *Sunu jing* ), an early Chinese text on sexual cultivation, ‘in preparation for their trysts’.^73^ In sexual cultivation, the intercourse of woman and man is understood as the harmonisation of *yin*–*yang*, water–fire, essence*–* *qi* , dragon–tiger, *kan* – *li* , mercury–lead, mouth–perineum, and the *Ren* *–* *Du* vessels, reflecting the Microcosmic Orbit principles underlying the practice of quiet sitting as well.

## *Qigong* as evidence for *Jingluo*

During the Long March and the second phase of the Chinese Civil War, the CCP’s army incorporated acupuncture into its medical repertoire for treating battlefield injuries and providing general healthcare. Influenced by Japanese acupuncture theories, which argued that ‘without eliminating the *jingluo* framework from acupuncture, it is difficult for acupuncture to attain scientific validity’, CCP acupuncturists Lu Zhijun and Zhu Lian (1909–1978) avoided employing the traditional *jingluo* framework in their clinical practice, instead equating *jingluo* with the nervous system.^74^ Beginning in 1948, in the CCP’s Southern Hebei Administrative Office, where Lu Zhijun worked from 1945 to 1947, Liu Guizhen (1920–1983) combined a local Inner-Nourishing Cultivation ( *neiyang gong* ) with the method of quiet sitting, thereby creating what later came to be known as *qigong* .^75^

In the early years of the PRC, the close Sino-Soviet relationship played a pivotal role in shaping national development.^76^ The outbreak of the Korean War (1950–1953) further deepened China’s dependence on the Soviet Union, sparking a nationwide trend of ‘Sovietization’. Soviet Pavlovian neuropathology and neurophysiology emerged as the dominant theoretical framework within Chinese medical science. Acupuncture, recast as part of China’s cultural heritage that could be explained through Pavlovian theory and endorsed by Soviet medical experts, gained strong political and institutional support.^77^ In 1953, following the Korean War armistice, the death of Joseph Stalin (1878–1953), the preliminary completion of economic recovery, and the political purge of Gao Gang (1905–1954) and his subordinates, particularly within the Ministry of Health, Mao shifted his attention to the medical field. As a nationalistic Communist,^78^ Mao sought to elevate the global status of Chinese medicine. On 4 June 1954, Mao ordered the establishment of a national research institute for Chinese medicine and directed ‘Western-trained physicians should study Chinese medicine’.^79^

Under these circumstances, in October 1954, Jiang’s *Yinshizi’s Method of Quiet Sitting*, both the main and supplementary volumes, became the first Republican-era *qigong* works to be reprinted in the PRC.^80^ The first newly compiled *qigong* book published in the PRC was Jiang’s *Yinshizi’s Discussion on Quiet Sitting and Hygiene Experiments* (*Yinshizi* *jingzuo weisheng shiyantan* ), interpreted in terms of Pavlovian theory, and published in October and December 1954 by two different presses.^81^ In this book, Jiang reflected that ‘Because I had long cultivated the practice of quiet sitting, the central vessel running from the perineum to the crown of the head had already been opened’.^82^ He further claimed:

Up to the present, another thirty-six years have passed [since the publication of *Yinshizi’s Method of Quiet Sitting*] (I am now eighty-two), and I have accumulated considerable experience, confirming the pathways of the Eight Extraordinary Vessels, which can serve as valuable references for both medical treatment and disease prevention.^83^

Jiang’s verification of *jingluo* provided precisely the kind of evidence addressing the perceived lack of scientific grounding in Chinese medicine, an issue frequently raised by Western-trained physicians, and it arrived at a moment when such support was urgently needed for Mao’s ‘Western-trained physicians should study Chinese medicine’ campaign, officially launched nationwide in November 1954.^84^

On 15 April 1955, during a dinner with Zhu Lian, Mao expressed his expectation that ‘the study of acupuncture will have an enormous impact on the reform of medical theory’. The author suggests that what Mao placed his confidence in was the uncovering of acupuncture’s theoretical basis, specifically the substantiation of the existence of *jingluo* , which had the potential to reconstruct the foundations of modern biomedicine. After offering a toast for ‘Long live acupuncture’, Mao added, ‘Do not assume that acupuncture is some backward folk remedy. Acupuncture is not backward; it is scientific, and in the future, countries all over the world will utilise it’.^85^ In May 1955, Cheng Danan (1899–1957), Principal of the Jiangsu Provincial School for Advanced Studies in Chinese Medicine ( *Jiangsusheng zhongyi jinxiu xuexiao* ), whom Mao had personally received and praised in December 1954 as ‘a highly renowned acupuncturist’, began urging the teachers and students at his school to initiate the study of *jingluo* theory.^86^ Like Jiang Weiqiao, Cheng also suffered from spermatorrhoea, was a devoted practitioner of *qigong*, and had previously practised quiet sitting cultivation at the Tongshan Society, an association well known to Jiang and visited by him.^87^ After the founding of the PRC, however, Cheng had aligned himself closely with Zhu Lian and Ivan Pavlov, avoiding any reference to *jingluo* in both his own teaching and in his school’s curriculum until this turning point. Under Cheng’s leadership, *jingluo* theory began to be incorporated into his school’s acupuncture curriculum beginning in May 1955.^88^

On 19 December 1955, the China Academy of Chinese Medicine was established, where acupuncturists Lu Zhijun served as director and Zhu Lian as deputy director. In its inauguration ceremony, Liu Guizhen’s *qigong* research team was formally acknowledged and commended for its major contributions to the nation’s medical heritage.^89^ Beginning in March 1956, Jiang began teaching *qigong* at several institutions.^90^ From that point until shortly before his death in 1958, numerous high-ranking Party members received *qigong* therapy from him. In his post-1949 teachings, Jiang promoted not only quiet sitting but also external physical exercise, such as Tai Chi.^91^ In July and August 1956, he was invited to present his *qigong* method at the China Academy of Chinese Medicine and Liu Guizhen’s Beidaihe Qigong Sanatorium.^92^ In these lectures, Jiang introduced his achievement of unobstructed circulation through the *Ren–Du* vessels, ‘a continuous cycle flowing from back to front’, as well as the opening of the Eight Extraordinary Vessels. His lecture at the Academy was subsequently published in the *Journal of Chinese Medicine* ( *Zhongyi zazhi* ) and later issued by the People’s Health Publishing House.^93^

In February 1956 and again in January 1957, Cheng proposed that *jingluo* ‘constitutes one of the fundamental theories of Chinese medicine’, and should therefore be studied and researched.^94^ In early 1957, *jingluo* theory was incorporated for the first time into a Chinese medicine textbook compiled by Cheng’s school.^95^ This volume later served as the prototype for the first national Chinese medicine textbook, *An Introduction to Traditional Chinese Medicine* ( *Zhongyixue gailun* ), published in Beijing in September 1958 and adopted as compulsory reading for Western-trained physicians, Chinese medicine practitioners, and acupuncturists nationwide.^96^ In April 1957, Cheng further argued that the origins of *jingluo* might lie in *qigong* practice:

The discovery of *jingluo* may well have originated from *qigong* . This is because, in the course of practising *qigong* , one can personally experience the sensation of opening the *Ren* and *Du* vessels. Once these vessels are opened, the practitioner is able to strengthen the body, prolong life, and treat illness. Thus, the integration of *jingluo* with medical practice is entirely natural. At the same time, since *jingluo* corresponds to physiological phenomena manifested during *qigong* practice, what ‘mystery’ or ‘superstition’ is there to speak of? The invention of acupuncture arose from accumulated experiential struggles against illness in daily life, and at the beginning, it could not possibly have been guided by any established theoretical framework. Only by gradually correlating *jingluo* phenomena with clinical experience did practitioners progressively develop and establish a complete and systematic *jingluo* theory to serve as the theoretical foundation for practice.^97^

Based on Cheng’s diary, the author argues that Cheng himself never succeeded in opening his *jingluo* through *qigong* practice. Jiang, for his part, claimed only to have opened his Eight Extraordinary Vessels, never the twelve primary *jing* or the fifteen *luo* that constitute the principal *jingluo* system described in the transmitted acupuncture classics, including *Nan* *jing* , *Zhenjiu jiayi* *jing* , *Huangdi nei* *jing* *taisu*, *Suwen*, and *Lingshu.* Moreover, the excavated *mai* manuscripts contain no references to the Eight Extraordinary Vessels, and in all of the transmitted texts listed above, the flow of the Eight Extraordinary Vessels is depicted as centripetal, therefore cannot form the continuous circulatory pathway envisioned in the Microcosmic Orbit.

However imprecise these claims of *jingluo* ‘verification’ may have been, the *jingluo* system was incorporated into acupuncture textbooks and firmly established as the discipline’s core theoretical foundation. Beginning in 1957, it became institutionalised as a foundational epistemological pillar of Chinese medicine through the construction of basic theory textbooks, a process led primarily by Neo-Confucian scholar-physicians, acupuncturists, and *qigong* practitioners, particularly those based in Shanghai and Jiangsu.^98^ With the onset of the Great Leap Forward (late 1957–early 1960), national experimental research on the physiological nature of *jingluo was* launched. This research later facilitated the widespread application of acupuncture anaesthesia during the Cultural Revolution, which in turn sparked an ‘acupuncture fever’ in Euro-American countries following Henry Kissinger’s 1971 visit to China, and ultimately contributed to the nationwide rise of *qigong* fever in the post-Mao era.^99^

## Conclusion

Long overlooked, Mao Zedong’s engagement with quiet sitting was not a youthful eccentricity but part of a broader culture of meditative practice shared by many contemporary elites, including Jiang Weiqiao, Yang Changji, Cai Hesen, Zhang Kundi, Xie Juezai, Xu Fanting, and Cheng Danan. By reconstructing this intellectual lineage, this article demonstrates that *qigong* fever was not a sudden post-Mao phenomenon. Rather, it was the cumulative outcome of a long and layered history in which Buddhist meditation, Daoist internal alchemy and sexual cultivation, Neo-Confucian self-cultivation and health preservation, and especially the Microcosmic Orbit circulation of the *Ren–Du* vessels intersected, were reinterpreted through modern foreign scientific discourses, and were ultimately consolidated in the 1950s through the state-supported verification, legitimation, and institutionalization of *jingluo* theory. These developments collectively shaped the historical transformation of *jingluo* from blood vessels to the nervous system, and finally to pathways of *qi*, thereby laying the epistemic groundwork for the *qigong* fever.

*Qigong*, whether in the form of meditative quiet sitting or coordinated bodily movements, historically circulated among Buddhist, Daoist, and Neo-Confucian elites, and never formed part of the therapeutic repertoire through which grassroots herbalists, acupuncturists, and other low-ranking healers, most of whom practised in rural areas, earned their livelihoods. In the 1950s as well, those who received *qigong* therapy from Jiang Weiqiao, Liu Guizhen, and other *qigong* masters were almost exclusively privileged CCP officials. Owing to the long-standing social division separating *qigong* practitioners from ordinary laypeople, including the vast majority of Chinese medicine practitioners and acupuncturists, the two groups had virtually no points of contact prior to the post-Mao reforms.

In Maoist China, Chinese medicine practitioners and acupuncturists were elevated to positions of authority and tasked with instructing biomedicine doctors. The construction of Chinese medicine’s basic theories came to be dominated by Neo-Confucian physicians, acupuncturists, and *qigong* practitioners, and was built upon the epistemological foundations of the circulatory *jingluo* system. Through successive political movements from the 1950s to the end of the Cultural Revolution, *jingluo* theory came to be widely accepted by the Chinese population as scientific truth. In the post-Mao reform era, *qigong*, once an elite and politically privileged practice, became accessible to the general public and rapidly gained nationwide popularity.

## Footnotes

1

David A. Palmer , Qigong Fever: Body, Science, and Utopia in China (New York: Columbia University Press, 2007), 6.

2

Guo Qingfan , Zhuangzi jishi (Beijing: Zhonghua shuju, 1961), 535 . Livia Kohn , Chinese Healing Exercises: The Tradition of Daoyin (Honolulu: University of Hawai‘i Press, 2008).

3

Joseph Needham and Lu Gwei-Djen , Science and Civilisation in China (Cambridge: Cambridge University Press, 1983), vol. 5, part 5, 2. Zhong Liquan and Lu Dongbin , Lingbao bifa (Taiyuan: Shanxi renmin chubanshe, 1990), 13, 23, 25. Zhang Junfang and Li Yongsheng (eds), Yunji qijian (Beijing: Zhonghua shuju, 2003), 949–1004 .

4 Shao Yong, ‘*Yinchuan jirang ji*’, in Guo Yu and Yu Tianbao (eds), *Shaoyong quanji* (Shanghai: Shanghai guji chubanshe, 2015), 75, 101, 148, 213, 222, 247, 266, 270, 271. Cheng Hao and Cheng Yi, ‘*Ercheng ji*’, in Wang Xiaoyu (ed.), *Ercheng ji* (Beijing: Zhonghua shuju, 1981), 50, 98, 202, 430, 432. Zhu Xi, *Zhuzi quanshu* (Shanghai: Shanghai guji chubanshe, 2002), 628, 1786, 2018, 2143, 2213, 2381, 2517, 3635, 3659. Wang Shouren, *Wang Yangming quanji* (Hangzhou: Zhejiang guji chubanshe, 2010), 12, 17, 62, 100–101, 114–5.

5

Nancy N. Chen , Breathing Spaces: Qigong, Psychiatry, and Healing in China (New York: Columbia University Press, 2003). Palmer, *op. cit.* (note 1).

6 Palmer, *op. cit.* (note 1), 45. Zhou Yuanchao, ‘Lun Yang Changji xiansheng dui Mao Zedong zaoqi tiyu sixiang de yingxiang’, *Dangdai jiaoyu lilun yu Shijian*, 1, 1 (2009), 3–4.

7 Gao Shanshan, ‘Forging a Basic Theory of Chinese Medicine in the Early People’s Republic of China: An Examination of *Wuxing*’ (unpublished PhD thesis: City University of Hong Kong, 2024).

8

Jiang Weiqiao , Yinshizi jingzuofa (Beijing: Xinshijie chubanshe, 2016), 2–4 . Jiang Weiqiao, *Zhuweng ziding nianpu* (n.p., n.d.) vol. 1, 23.

9

Jiang Weiqiao , Yinshizi jingzuofa (Shanghai: Shangwu yinshuguan, 1927), 32 . Jiang Weiqiao and Zhang Zanchen , Yinshizi jingzuo weisheng shiyantan (Shanghai: Shanghai zhongyi shuju, 1954), 28–9.

10

Wang Ang , Yifang jijie (Kyoto: Yoshinoya Hakubundō, 1727).

11

Jiang Weiqiao , Yinshizi jingzuofa (Shanghai: Shangwu yinshuguan, 1927), 33 .

12

Jiang Weiqiao , Lin Pan , Hu Xinxuan , and Wang Weidong (eds), Jiang weiqiao riji (Shanghai: Shanghai renmin chubanshe, 2021), vol. 1, 36 . Jiang Weiqiao , Yinshizi jingzuofa (Shanghai: Shangwu yinshuguan, 1927), 36 . Jiang Weiqiao and Zhang Zanchen , Yinshizi jingzuo weisheng shiyantan, 29–30 . Jiang, *op. cit.* (note 8).

13
*Ibid.*, Jiang et al., *Jiang weiqiao riji*, vol. 2, 625.

14

Jiang Weiqiao , Yinshizi jingzuofa (Shanghai: Shangwu yinshuguan, 1914).

15 Jiang, *op. cit.* (note 8).

16 In 1915, Jiang published his *Theory of Maintaining Eternal Health and Youth Through the Elimination of Breakfast*, a revised version of Mishima Kin’ichirō’s Japanese work. See: Mishima Kin’ichirō , Theory of Maintaining Eternal Health and Youth Through the Elimination of Breakfast and Adopting a Two-Meal (Tokyo: Kaihatsu, 1914). Jiang Weiqiao , Jiankang bulao feizhi zhaoshi lun (Shanghai: Shangwu yinshuguan, 1915).

17 Chi-Yang Liao, ‘Building Modernity Based on Internal Evidence and a View Encompassing East Asia, Along With the Criticism Thereof: Jiang Wei-qiao and the Okada-style Seiza Meditation’, *Siyuyan*, 59, 1 (2021), 99–154. Hsiu-fen Chen, ‘Treating Disease, Strengthening the Body and Reforming Nationality: Jiang Weiqiao’s Meditative Sitting and the Body Experiences in Early Republican China’, *Xin shixue*, 34, 1 (2023), 95–152.

18

Xiao Yu , Mao Zedong: qingnian shidai (Hong Kong: Mingbao youxian gongsi chubanbu, 1977), 47 ; Luo Shaozhi and Luo Xinhu , ‘Mao Zedong yu He Guo’, in Dai Baihan (ed.), Qingnian Mao Zedong yu tade xiangji shiyou (Changsha: Hunan chubanshe, 1993), 238 ; Li Li , Mao Zedong hetade tongxuemen (Beijing: Tuanjie chubanshe, 2017), 178 .

19

Jiang Weiqiao and Zhang Zanchen , Yinshizi jingzuo weisheng shiyantan, 31 . Yinshizi, *Yinshizi jingzuofa xubian* (Shanghai: Shangwu yinshuguan, 1922).

20 Jiang and Zhang, *op. cit.* (note 9), 32.

21 Jiang, *op. cit.* (note 14), 39–40.

22 Nobuta Kishimoto and Hua Wenqi (trans.), Jiang Weiqiao (collated, revised, and prefaced), *Jingzuo sannian* (Shanghai: Shangwu yinshuguan, 1916). Reisai Fujita and Liu Renhang (trans.), Jiang Weiqiao (collated, revised, and prefaced), *Shenxin tiaohefa* (Shanghai: Shangwu yinshuguan, 1916). Reisai Fujita and Liu Renhang (trans.), Jiang Weiqiao (prefaced), *Shenxin qiangjian mijue* (Shanghai: Shangwu yinshuguan, 1916). Kaiten Nukariya and Liu Renhang (trans.), Jiang Weiqiao (collated and revised), *Beimei yujia xueshuo* (Shanghai: Shangwu yinshuguan, 1918). Ema Shun’ichi and Amino Reihō, Jiangxia Yunhe (trans.), *Xinshen duanlianfa* (Shanghai: Shangwu yinshuguan, 1919). Tokujirō Okada and Jiang Weiqiao (trans.), *Gangtianshi jingzuofa* (Shanghai: Shangwu yinshuguan, 1919). Gosaku Hashimoto and Lei Tongqun (trans.), Jiang Weiqiao (prefaced), *Gangtianshi jingzuo xinli* (Shanghai: Shangwu yinshuguan, 1920).

23

Liu Renhang , Letian quebing fa (Shanghai: Shangwu yinshuguan, 1915). Zhou Mingze , Zhou Zhongfang jingzuofa mishu (Shanghai: Shangwu yinshuguan, 1916). Ding Fubao , Jingzuofa jingyi (Shanghai: Yixue shuju, 1920). Hunyizi , Wuwei jingzuofa (Shanghai: Chongdao lianyishe, 1925). Lin Runhan , Jianshen shoushi (Beiping: Guomin jiankang kuaile zhinanshe, 1933). Xiao Ping , Quebing yannian changshengshu (Shanghai: Dazhong shuju, 1933). Xiao Changming , Jingshen zhiliao jieshuo (Beijing: Beijing zongjiao zhexue yanjiushe, 1940). Yang Jianxing , Zhidao zhenquan (Shanghai: Rongwu jiangjingshe, 1941). Liu Youzhong , Chongyong jingzuoxue (Shanghai: Laozhuang jingshe, 1949).

24 Li Duanshi, *Jiuyishi jingshen zhiliao yishu ershida xinfa* (n.p., Guomin jingshen yangchenghui liaoyangyuan, 1926). Yang Daozhen , Jingshen ganying zhiliaofa (Beiping: Yang Daozhen jingshen zhiliaosuo, 1929). Wei Hongsheng , Jingshen bingyuan wenda (Beiping: Beiping jingshen bingyuan, 1933). Fu Jiankang , Jingshen ganying zhiliaofa (Tianjin: Fu Jiankang jingshen zhiliaosuo, 1935). Wei Hongsheng , Jingshen shiyan wenda (Beiping: Hongsheng zhiliaoyuan, 1936). Wei Hongsheng , Jingshen jiankang shiyanlu (Beiping: Wei Hongsheng zhiliaoyuan, 1937). Wei Hongsheng , Gaodeng jingshen duanlian zhiliao wenda (Beiping: Wei Hongsheng zhiliaoyuan, 1937).

25 Palmer, *op. cit.* (note 1), 50–51. Vincent Goossaert and David A. Palmer , The Religious Question in Modern China (Chicago: The University of Chicago Press, 2011), 91–121 . Wang Chien-chuan and David Ownby (trans.), ‘The composition and distribution of the scriptures of the Tongshanshe, with a focus on the *Ten Thousand Buddha Scripture* (1917–1949)’, in Philip Clart , David Ownby , and Wang Chien-chuan (eds), Text and Context in the Modern History of Chinese Religions: Redemptive Societies and Their Sacred Texts (Leiden: Brill, 2020), 55–81 .

26

Liang Shuming , Liang Shuming huiyilu (Beijing: Beijing wenshi chubanshe, 2012), 51, 125, 182.

27

Edgar Snow , Red Star Over China: The Classic Inside Account of Mao’s China (New York: Grove Press, 1978), 133 .

28
*Ibid.*, 133, 140.

29

Mao Zedong , ‘Zhiyourenxin (1915.7)’, in Zhonggong zhongyang wenxian yanjiushi, Zhonggong hunan shengwei Mao Zedong zaoqi wengao banji zu (eds.), Mao Zedong zaoqi wengao (1912.6–1920.11) (Changsha: Hunan chubanshe, 1990), 14 . Cao Dianqiu , ‘Yang Cahngji xianshengzhuan’, in Wang Xingguo (ed.), Yang Cahngji wenji (Changsha: Hunan jiaoyu chubanshe, 1983), 386 .

30

Harrison E. Salisbury , The Long March: The Untold Story (New York: Harper & Row, 1985), 76 .

31 Zhang Shizhao, ‘Yang Huaizhong biezhuan’, in Wang Xingguo (ed.), *Yang Cahngji wenji*, 388–9.

32 Yang Changji, *Dahuazhai riji (jiaodingben)* (Changsha: Hunan renmin chubanshe, 1981), 96.

33 1896, 1899, 1914, 1915, and 1919.

34 Yang, *op. cit.* (note 32), 87.

35 Liyan Liu, ‘The Man Who Molded Mao: Yang Changji and the First Generation of Chinese Communists’, *Modern China*, 32, 4 (2006), 502.

36 Yang, *op. cit.* (note 32), 119–22.

37
*Ibid.*, 119, 122.

38 Li Xiaoran, ‘Benxiao gujiaoshou yanghuaizhong xiansheng shiji’, in Wang Xingguo (ed.), *Yang Changji wenji*, 375–376.

39 Yang, *op. cit.* (note 32), 48.

40 Jiang Weiqiao, *Yinshizi jingzuofa* (Shanghai: Shangwu yinshuguan, 1927), 11, 16, 21–7, 34.

41 Zhonggong zhongyang wenxian yanjiushi, *Mao Zedong nianpu (1893–1949)* (Beijing: Zhongyang wenxian chubanshe, 1993), 29.

42 Edgar Snow translated it into ‘The Energy of the Mind’.

43 Snow, *op. cit.* (note 27), 133.

44 Zhonggong zhongyang wenxian yanjiushi, *op. cit.* (note 41), 31.

45 Snow, *op. cit.* (note 27), 135.

46 Yang’s courtesy name.

47 Xiao San, *Mao Zedong tongzhi de qingnian shidai* (Beijing: Renmin chubanshe, 1949), 61–2.

48 Tang Chunyuan, ‘Wenge shiqi maozedong shengping zhuanji de wenben yanjiu’, *Hunan keji daxue xuebao (shehui kexueban)*, 14, 2 (2011), 21–2.

49 Mao and Cai enrolled in the same class at the First Normal in the spring of 1914, and soon developed a close friendship. Both became dedicated disciples of Yang. Cai began practicing *Yinshizi’s Method of Quiet Sitting* in the autumn of 1915. During the summer break of 1917, neither Cai nor Zhang was in Mao’s company. Yang and Mao both relocated to Beijing in the summer of 1918. Taken together, this timeline strongly suggests that the summer of 1916 was the most plausible period in which Mao engaged in quiet sitting practices. See: Zhonggong shuangfengxian xuanchuanbu (ed.), *Huainian Cai Hesen tongzhi* (Changsha: Hunan renmin chubanshe, 1980), 3, 52, 54, 57–8. Li Rui, *Sanshisui yiqiande maozedong* (Guangzhou: Guangdong renmin chubanshe, 1994), 184, 305.

50 Wang Guangxia, ‘Liaoyuan biyi’, in Zhonggong shuangfengxian xuanchuanbu (ed.), *Huainian Cai Hesen tongzhi*, 58.

51 Zhang Kundi, ‘Zhang Kundi’s diary’, in Li Rui, *Sanshisui yiqiande maozedong* (Guangzhou: Guangdong renmin chubanshe, 1994), 184.

52 Xiao San, ‘Mao Zedong tongzhi de qingnian shidai’, *Shidai qingnian* (n.p., 1946–1947).

53 Xiao San, *Mao Zedong tongzhi de qingnian shidai* (n.p., Dongbei shudian, 1947). Xiao San, *Mao Zedong tongzhi: ertong shidai qingnian shidai yu chuqi geming huodong* (n.p., Shandong xinhua shudian, 1947).

54 See Zhongguo renmin daxue tushuguan, *Jiefangqu genjudi tushu mulu* (Beijing: Zhongguo renmin daxue chubanshe, 1989), 86–90.

55 Xiao San, *Mao Zedong tongzhi de qingnian shidai* (n.p., Dongbei shudian, 1947); Xiao San, *Mao Zedong tongzhi: ertong shidai qingnian shidai yu chuqi geming huodong* (n.p., Shandong xinhua shudian, 1947); Xiao San, *Mao Zedong tongzhi de qingshaonian shidai* (n.p., Xinhua shudian, 1949); Xiao San, *Mao Zedong tongzhi de qingshaonian shidai* (Beijing: Renmin chubanshe, 1949); Xiao San, *Mao Zedong tongzhi: ertong shidai qingnian shidai yu chuqi geming huodong* (Wuxi: Sunan xinhua shudian, 1949); Xiao San, *Mao Zedong tongzhi de qingshaonian shidai* (Beijing: Renmin chubanshe, 1950).

56 Xinxiang pinglun bianjibu, *Mao Zedong tongzhi de qingshaonian shidai* (Beijing: Zhongguo qingnian chubanshe, 1979); Xinxiang pinglun bianjibu, *Mao Zedong tongzhi de qingshaonian shidai* (Changsha: Hunan renmin chubanshe, 1979); Xiao San, *Mao Zedong tongzh de qingshaonian shidai he chuqi geming huodong* (Beijing: Zhongguo qingnian chubanshe, 1980).

57

Mao Zedong , ‘Tiyu zhi yanjiu (1917.4.1)’, in Zhonggong zhongyang wenxian yanjiushi, Zhonggong hunan shengwei Mao Zedong zaoqi wengao banji zu (eds.), Mao Zedong zaoqi wengao (Changsha: Hunan chubanshe, 1990), 65 .

58
*Ibid.*, 66.

59
*Ibid.*, 69.

60
*Ibid.*, 74–5.

61
*Ibid.*, 75–6.

62
*Ibid.*, 76–8.

63 Wang, *op. cit.* (note 50), 58.

64 Hong Mai, *Yijianzhi* (Beijing: Zhonghua shuju, 1981), 258–9.

65 Given the limitations of the available evidence, it remains unclear why Mao emphasised *luo* and *xuemai* while making no explicit reference to *jing* (channels). One possible explanation is that the character *jing* also carries the meaning of ‘classics’, a term with associations that Mao, as a revolutionary, may have found ideologically unappealing. Moreover, *jing* is homophonous with terms denoting reverence, stillness, and quiet sitting, the very dispositions he was criticising in the article.

66 ‘Zubi shiyimai jiujing’, ‘Yinyang shiyimai jiujing jiaben’, in Qiu Xigui (ed.), *Changsha mawangdui hanmu jianbo jicheng* (Beijing: Zhonghua shuju, 2014), vol. 5, 187–204. ‘Yinyang shiyimai jiujing yiben’, in Qiu Xigui (ed.), *Changsha mawangdui hanmu jianbo jicheng* (Beijing: Zhonghua shuju, 2014), vol. 6, 9–13. ‘Maishu’, in Zhangjiashan 247 hao handu zhujian zhengli xiaozu (ed.), *Zhangjiashan hanmu zhujian (247 haomu)* (Beijing: Wenwu chubanshe, 2006), 118–24. ‘Shier jingmai’, in Tianhui yijian zhenglizu (ed.), *Tianhui yijian* (Beijing: Wenwu chubanshe, 2022), 41–7.

67 Huangfu Mi (ed.), ‘*Zhenjiu jiayijing*’, in *Dongyang yixue shanben congshu* (Osaka: Orient Publishing House, 1978), vol. 7, 9, 11. Wang Bing (ed.), ‘*Lingshu jing*’, in *Dongyang yixue shanben congshu*, vol. 26, 114. Yang Shangshan , Li Yun (ed.), Riben jingdu renheshi yuancang guchaoben huangdi neijing taisu xinjiao (Beijing: Xueyuan chubanshe, 2019), 75 .

68 Gao, *op. cit.* (note 7), 20–26.

69 Snow, *op. cit.* (note 27), 135.

70 Mao, *op. cit.* (note 57), 69–77.

71

Xie Juezai , Xie Juezai riji (Beijing: Renmin chubanshe, 1984), 1044 . Xu Lei , ‘Funujian: huainian wode fuqin Xu Fanting’, in Xing Tongke (ed.), *Xu Fanting jiniance* (Beijing: Huawen chubanshe, 1995), 189 .

72 Shanxi weishengzhi bianzuan weiyuanhui bangongshi, *Shanganning bianqu yijia zhuanlue* (Xian: Shanxi kexue jishu chubanshe, 1991), 149. Qin Qiu, ‘Mao Zedong guanxin zhongxiyi jiehe shiye: cong Lu Zhijun wei Mao zhuxi zhibing tanqi’, *Jiating zhongyiyao,* 12 (1993), 4.

73

Li Zhisui , The Private Life of Chairman Mao (New York: Random House, 1994), 68, 358.

74 Lu Zhijun, *Zhenjiuxue* (Dabieshan: Zhongguo renmin jiefangjun zhongyuan junqu weishengbu, 1949). Lu Zhijun, *Xinbian zhenjiuxue* (Chongqing: Chongqing renmin chubanshe, 1950). Zhu Lian, *Xin zhenjiuxue* (Beijing: Renmin chubanshe, 1951). Gao Zengjie (trans.), ‘Riben xuezhe zuotan jingluo wenti’, in Hunansheng zhenjiu xuehui, Hunan zhongyi xueyuan zhenjiu jiaoyanshi (eds.), *Zhenjiu ziwu liuzhu xuanzhai* (n.p., 1981), 33–41.

75

Liu Guizhen , Qigong liaofa shijian (Hebei: Hebei renmin chubanshe, 1957), 65 . Liu Guizhen , Qigong liaofa (Beidaihe: Hebeisheng beidaihe liaoyangyuan, 1956), 2 .

76

Oleg Borisovich Borisov and B. T. Koloskov , Soviet-Chinese Relations, 1945–1970 (Bloomington: Indiana University Press, 1975), 58–9.

77 Gao Shanshan, ‘Tikhon Efimovich Boldyrev (1900–1984): A Soviet Epidemiologist’s Contributions to Public Health in 1950s China’, *Journal of Medical Biography.* https://journals.sagepub.com/doi/10.1177/09677720251398961. First published online 21 November 2025.

78

Joseph Levenson , Revolution and Cosmopolitanism: The Western Stage and the Chinese Stages (Berkeley: University of California Press, 1971), 27 .

79 Hu Xiaofeng and Li Aijun, ‘Zhongguo zhongyi kexueyuan dashiji’, *Zhonghua yishi zazhi*, 45, 6 (2015), 361.

80 Jiang Weiqiao and Zhang Zanchen (eds), *Yinshizi jingzuofa zhengbian (dingzhengben)* (Shanghai: Shanghai caiyun yuxie youyinshe, 1954). Jiang Weiqiao and Zhang Zanchen (eds), *Yinshizi jingzuofa xubian (dingzhengben)* (Shanghai: Shanghai caiyun yuxie youyinshe, 1954).

81 Also titled *Chinese Methods of Medical Treatment and Disease Prevention*, see Jiang Weiqiao and Zhang Zanchen (eds), *Yinshizi jingzuo weisheng shiyantan (yiming zhongguo yiliao yufangfa)* (Shanghai: Shanghai caiyun yuxie youyinshe, 1954). Jiang Weiqiao and Zhang Zanchen (eds), *Yinshizi jingzuo weisheng shiyantan (yiming zhongguo yiliao yufangfa)* (Shanghai: Shanghai zhongyi shuju, 1954).

82 Jiang Weiqiao and Zhang Zanchen, *Yinshizi jingzuo weisheng shiyantan*, 41.

83
*Ibid.*, 2.

84 Zhonggong zhongyang, ‘Zhonggong zhongyang pizhuan zongyang wenwei dangzu guanyu gaijin zhongyi gongzuo wenti de baogao (1954.11.23)’, in Zhonghua renmin gongheguo weishengbu zhongyisi (ed.), *Zhongyi gongzuo wenjian huibian (1949–1983)* (Beijing: Zhonghua renmin gongheguo weishengbu zhongyisi, 1985), 43.

85

Mai Yang and Liu Peng , Mao Zedong zai 1958 (Beijing: Zhongguo qingnian chubanshe, 2008), 99–100 .

86

Cheng Danan , Cheng Danan yiji (Beijing: Zhongguo zhongyiyao chubanshe, 2017), vol. 2, 1916, 1946, 1979–80.

87
*Ibid.*, 1981. Jiang Weiqiao, *Jiang Weiqiao riji* (Beijing: Zhonghua shuju, 2014), vol. 7, 420. Fan Chun-wu, ‘Wusi shidai zhongguo shehui jingzuo fengchao’, in Lu Miaw-fen, Paul R. Katz (eds), *Wusi yundong yu zhongguo zongjiao de tiaoshi yu fazhan* (Taipei: Zhongyang yanjiuyuan jindaishi yanjiusuo, 2020), 305–6.

88 Cheng, *op. cit.* (note 86), 1980.

89

Hua Zhongfu and Liang Jun , Zhongguo zhongyi yanjiuyuan yuanshi (1955–1995) (Beijing: Zhongyi guji chubanshe, 1995), 8–9 .

90 Including the Shanghai Public Medical Clinic of Chinese Medicine, the PLA No. 87 Sanatorium, the Taihu Sanatorium in Wuxi, the Hongqiao Sanatorium in Shanghai, and the Yejia Garden Sanatorium. See: Jiang Weiqiao, *Jiang weiqiao riji* (Beijing: Zhonghua shuju, 2014), vol. 8, 4104–4129.

91 Jiang Weiqiao, *Zhongguo de huxi xijing yangshengfa (qigong fangzhi fa)* (Shanghai: Shanghai weisheng chubanshe, 1956), 9.

92 Jiang, *op. cit.* (note 90), 4135.

93 Jiang Weiqiao, ‘Tantan qigong zhiliaofa’, *Zhongyi zazhi*, 10 (1956), 557–9. Jiang Weiqiao and Liu Guizhen, *Zhongyi zhiliao jingyan xuanji: qigong liaofa* (Beijing: Renmin weisheng chubanshe, 1958), 1, 7, 8. Jiang, *op. cit.* (note 90), 4142–52.

94 Cheng Danan, ‘Jingluo de chubu renshi’, *Zhonghua yixue zazhi*, 42, 2 (1956), 134–7. Cheng Danan, ‘Guanyu zhenjiujie yinggai shouxian xuexi yanjiu jingluo xueshuo de yijian’, *Zhongyi zazhi*, 1 (1957), 24–5.

95 Jiangsusheng zhongyi xuexiao, *Zhongguo yixue gailun* (Nanjing: Jiangsusheng zhongyi xuexiao, 1957).

96 Nanjing zhongyi xueyuan, *Zhongyixue gailun* (Beijing: Renmin weisheng chubanshe, 1958).

97 Cheng Danan, ‘Jingluo wenti buneng cong jiepou de jiaodu qu lijie’, *Zhongyi zazhi,* 4 (1957), 200.

98 Gao, *op. cit.* (note 7), 93–217.

99 Xianshi yixue kexue yanjiusuo zhenjiu mazui yanjiushi, *Zhenjiu mazui* (Xian: Xian renmin chubanshe, 1959), 1.

